# Mapping and Ablation of Retrograde Conduction During a Nearly Incessant Pacemaker-mediated Tachycardia in a Patient with Third-degree Atrioventricular Block

**DOI:** 10.19102/icrm.2019.100402

**Published:** 2019-04-15

**Authors:** Valentino Ducceschi, Francesco Maddaluno, Laura Casaretti, Raffaele Sangiuolo

**Affiliations:** ^1^Pellegrini Hospital, Naples, Italy; ^2^Boston Scientific Italia, Milan, Italy; ^3^FateBeneFratelli Hospital, Naples, Italy

**Keywords:** Ablation, arrhythmia, atrioventricular block, pacemaker programming, pacemaker-mediated tachycardia

## Abstract

Patients with third-degree atrioventricular block implanted with a dual-chamber pacemaker in DDD mode can develop pacemaker-mediated tachycardias if retrograde ventriculoatrial (VA) conduction is present. Programming a long post-VA refractory period to avoid tachycardia initiation can be contraindicated if these patients have a good atrial response from exercise testing and require a high maximum tracking rate to allow for a proper response to sensed atrial rhythms. We report a case of a patient in whom mapping and ablation of retrograde conduction during the pacemaker-mediated rhythm was the only solution to allow both the programming of a high tracking rate and the elimination of tachycardia induction.

## Introduction

Antegrade and retrograde atrioventricular (AV) conduction through the AV node can occur completely independently of one another in individual patients. For this reason, patients with third-degree AV block can present with the paradoxical phenomenon of retrograde nodal conduction and develop pacemaker-mediated tachycardias (PMTs) if implanted with a dual-chamber pacemaker in DDD mode.^1^ When these patients show good atrial response following exercise testing, a high maximum tracking rate (MTR) is usually programmed to prevent a period of Wenckebach during physical exertion. The drawback of MTR programming is the risk of designating a PMT episode in the pacemaker memory, thus delaying its treatment, since only events with atrial-sensed activity and ventricular-paced rhythm with MTR can be recognized as PMT in some types of commercially available pacemakers.^2^ Without protection, PMTs can last for hours and even days, leading to severe symptoms and acute heart failure. If a good clinical compromise with pacemaker programming cannot be found, mapping and ablation of retrograde conduction during PMT is mandatory.

## Case presentation

**Figure 1:**
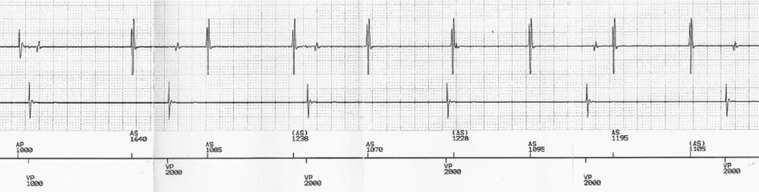
Temporary programming of the pacemaker to the stimulation VVI mode shows third-degree AV block.

A 60-year-old male patient with a dual-chamber pacemaker (Advantio™; Boston Scientific, Natick, MA, USA) implanted for third-degree AV block **(Figure 1)** presented to the emergency room with incessant wide-QRS tachycardia at around 135 bpm. This prolonged tachycardia (the patient referred to many episodes lasting hours) was very poorly tolerated and led to cardiac decompensation. Pacemaker interrogation revealed a ventricular-paced rhythm followed by atrial-sensed activity during tachycardia; thus, a PMT was suspected. Temporary programming of the pacemaker to simulate VVI mode led to arrhythmia termination, reinforcing the suspicion of PMT. However, no PMT episode was stored in the pacemaker memory because the device model only detects PMT during ventricular-paced rhythm with MTR. The MTR value had been previously programmed to 160 bpm because the patient’s atrial rate during physical exertion reached a level of more than 150 bpm.

**Figure 2:**
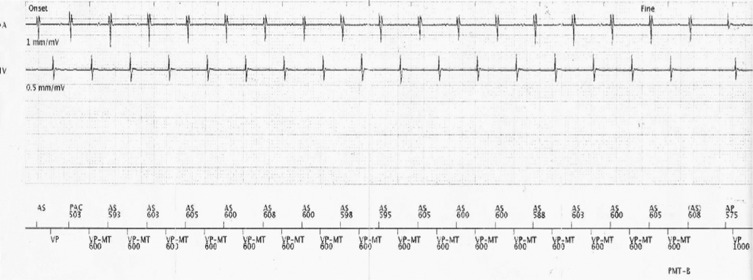
After the first atrial-sensed event, the programmed AV delay (200 ms) was applied before ventricular pacing. In the next beat, a PAC occurred and a longer AV interval was applied to avoid violating the MTR limit. After the ventricular-paced beat, the retrograde conduction depolarized the atrium, triggering a PMT that was interrupted by lengthening the PVARP after 16 paced beats.

To confirm the diagnosis of PMT and to determine the mechanism of induction, we lowered the MTR to 100 bpm so that some episodes could be stored. From the analysis of tracings, we found that the main reason for PMT induction was premature atrial contraction (PAC). **Figure 2** shows that a PAC is sensed by the atrial channel; however, the AV interval applied prior to ventricular pacing is slightly longer than the programmed AV interval applied on the previous beat. This occurs to avoid violating the MTR limit. This device algorithm enables the atrial tissue to recover so that retrograde conduction from the ventricular-paced beat depolarizes the atrium, giving rise to the PMT. This PMT is detected by the device and interrupted after 16 paced beats by prolonging the postventriculoatrial refractory period (PVARP) so that the retrograde atrial activation falls into this device refractory period without triggering ventricular pacing. The V-Ar-A-V response with modification of the atrial rate after termination confirmed the diagnosis of PMT.^3^

To prevent the retrograde atrium from being sensed and to avoid PMT induction, the PVARP should be programmed at 50 ms in excess of the ventriculoatrial (VA) conduction time.^1^ In this patient, the VA conduction time ranged from 270 ms to 280 ms. This value in combination with an AV interval of more than 160 ms would have limited the patient’s upper ventricular rate, with deleterious effects to cardiac function. For this reason, mapping and radiofrequency (RF) ablation of retrograde conduction during PMT were undertaken.

During the electrophysiological study, no atrial tachycardia could be induced by atrial pacing, whereas the clinical arrhythmia could easily be induced by right ventricular pacing. The clinical tachycardia showed a constant VA linking and the atrial rate suddenly decreased as soon as the ventricular pacing rate was lowered by pacemaker programming. This behavior ruled out the presence of focal atrial tachycardias and clearly showed that atrial activity during tachycardia was conducted retrogradely from the ventricle.

Pacing from the right ventricle revealed that VA conduction was concentric and decremental, ruling out the presence of accessory pathways.

**Figure 3:**
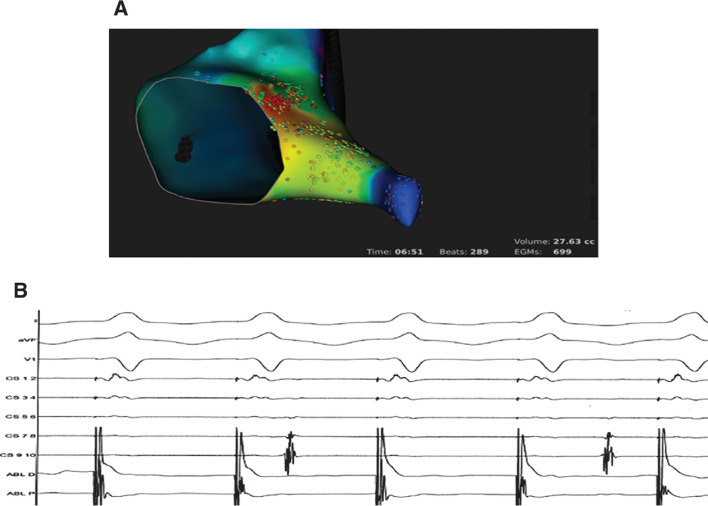
**A:** Rhythmia™ system (Boston Scientific, Natick, MA, USA) activation map (modified left anterior oblique view) showing the earliest atrial signal (red spot) in the His bundle region. **B:** No VA conduction during right ventricular pacing after RF ablation.

The site of earliest retrograde activation during PMT was mapped with the Rhythmia™ system (Boston Scientific, Natick, MA, USA) and the IntellaNav Mifi™ XP 8-mm-tip ablation catheter boasting miniature electrodes (Boston Scientific, Natick, MA, USA). The earliest atrial signal was found in the fast pathway region. A single RF application in this location interrupted the PMT, causing retrograde VA dissociation during right ventricular pacing **(Figure 3)**. At a follow-up consultation 12 months after the ablation procedure, no arrhythmia recurrence was recorded or reported by the patient.

## Discussion

Despite several programming options available on modern pacemakers, finding a combination of programmable values that allow for both high MTR and the prevention of PMT remains difficult. The most widespread solution to avoiding PMT is to increase the PVARP, but this option limits the patient’s upper ventricular rate and cannot be used where there is a good atrial response during exercise. This is because 2:1 AV block will occur during physical exertion. In addition, some patients can still have PMTs despite long values of programmed PVARP, especially during long VA conduction that leads to slow tachycardias.

Mapping and ablation of retrograde conduction during PMT is an important solution that should be considered even in patients with third-degree AV block when no alternative pacemaker algorithm can deliver improvement.
